# Telomeric G-Quadruplexes: From Human to* Tetrahymena* Repeats

**DOI:** 10.1155/2017/9170371

**Published:** 2017-12-28

**Authors:** Erika Demkovičová, Ľuboš Bauer, Petra Krafčíková, Katarína Tlučková, Petra Tóthova, Andrea Halaganová, Eva Valušová, Viktor Víglaský

**Affiliations:** ^1^Department of Biochemistry, Institute of Chemistry, Faculty of Sciences, P. J. Šafárik University, 04001 Kosice, Slovakia; ^2^Department of Biological Sciences/RNA Institute, University at Albany, SUNY, Albany, NY 12222, USA; ^3^Institute of Experimental Physics, Slovak Academy of Sciences, Watsonova 47, 040 01 Košice, Slovakia

## Abstract

The human telomeric and protozoal telomeric sequences differ only in one purine base in their repeats; TTAGGG in telomeric sequences; and TTGGGG in protozoal sequences. In this study, the relationship between G-quadruplexes formed from these repeats and their derivatives is analyzed and compared. The human telomeric DNA sequence G_3_(T_2_AG_3_)_3_ and related sequences in which each adenine base has been systematically replaced by a guanine were investigated; the result is* Tetrahymena* repeats. The substitution does not affect the formation of G-quadruplexes but may cause differences in topology. The results also show that the stability of the substituted derivatives increased in sequences with greater number of substitutions. In addition, most of the sequences containing imperfections in repeats which were analyzed in this study also occur in human and* Tetrahymena* genomes. Generally, the presence of G-quadruplex structures in any organism is a source of limitations during the life cycle. Therefore, a fuller understanding of the influence of base substitution on the structural variability of G-quadruplexes would be of considerable scientific value.

## 1. Introduction

G-rich DNA sequences can form intra- and intermolecular G-quadruplexes based on the association of one or more DNA strands. The nucleotides which intervene between G-runs form loops of folded G-quadruplex structures which can adopt a variety of different topological forms [1, 2]. When the guanine tracts are oriented in the same direction, the double-chain reversal (propeller) loops link two adjacent parallel strands to form a parallel structure [3]. When the guanine tracts are oriented in opposite directions, the edgewise or diagonal loops link two antiparallel strands to form an antiparallel G-quadruplex [4]. In antiparallel hybrid or so-called (3 + 1) structures, a single strand is oriented in a different direction from the others [5–7]. A novel (3 + 1) type fold which has recently been described by Marušič et al. exhibits a conformation in which all three loop types occur in one conformation: edgewise, diagonal, and double-chain reversal loops [8]. In addition, intermolecular multimeric G-quadruplexes can be formed by the association of two or more strands [9].

These structures underline the high degree of G-quadruplex structural polymorphism, a phenomenon which is dependent on many different factors: the length and sequence of nucleic acid, and environmental conditions present during the folding reaction such as the buffer, pH, stabilizing cation, temperature, and the presence of agents causing dehydration [10–14]. G-rich sequences with the propensity to form G-quadruplex structures can be located in many regions of human genomic DNA, especially in several biologically important regions including the end of linear eukaryotic telomeres [15, 16]. However putative G-rich sequences are not randomly distributed within a genome; such sequences predominantly occur in protooncogene regions (which promote cell proliferation) and are depleted in tumour suppressor genes (which maintain genomic stability) [17]. It is very unlikely that these putative sequences can form* in vivo* and direct evidence of their existence in living cells is still a topic of discussion [18–20]. Undoubtedly, the most extensively studied G-quadruplex forming sequences are those located at the 3′-ends of human telomeres. Telomeric sequences and specialized nucleoprotein complexes which cap the ends of linear chromosomes are essential for chromosomal stability and genomic integrity [21–23]. Mammalian telomeres consist of tandem repeats of G-rich sequences, d(TTAGGG)_*n*_. Several kilobases of this sequence are double-stranded, but more than a hundred nucleotides remain unpaired and form single-stranded 3′-overhangs [24], a state which would provide favourable conditions for the formation of one or more G-quadruplexes* in vivo* [22]. The structure and stability of telomeres play a significant role in the development of cancer and cell aging [25, 26]. There is also evidence that telomeres serve as a type of biological clock, as telomere structures appear to become shorter with each successive cell cycle. In immortalized cells and in cancer cells, however, a telomerase is activated to maintain the length of the telomere by reelongating the telomeric sequence at the chromosome ends [27, 28]. G-quadruplexes formed by single-stranded human telomeric DNA have also been shown to inhibit the activity of telomerase [29], and this discovery has led to increased interest in the structures as attractive potential drug targets [30].

A broad range of studies of human telomeric G-quadruplexes have been carried out using a wide variety of different techniques [1]. To date, high-resolution structures of four distinct folding topologies with three G-tetrad layers have been identified for the four human telomeric repeats [1–7]. In addition, an additional structure consisting of only two G-tetrad layers has also been revealed which highlights the structural polymorphism of telomeric G-quadruplexes [31]. The structure of human telomeric DNA in crowded solutions has also been investigated by many authors [11], but this structure is likely to be a result of dehydration rather than molecular crowding [12, 32, 33]. The great variety of structures identified to date can also be attributed to the presence of flanking nucleotides outside the core sequence G_3_(T_2_AG_3_)_3_ and the concentration of ions and to the use of different experimental methods and conditions [1, 34].

**Figure 1:**
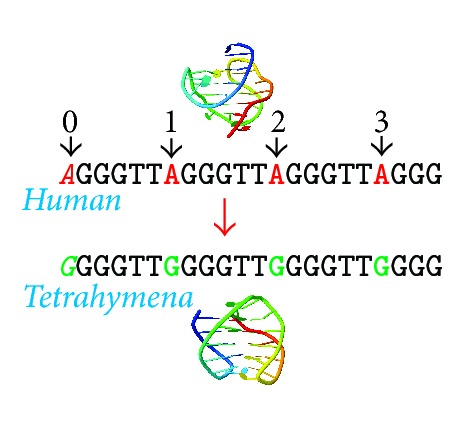
The HTR to THR conversion by substitutions of adenine for guanine.

A series of systematic studies concerning the sequence derivatives of human telomeric repeats were carried out by Vorlícková et al. [35–38], and these earlier studies focused on the substitution of guanine for adenine, the introduction of abasic sites, 8-oxoadenine replacing adenine, and the substitution of 5-hydroxymethyluracil for thymine in telomeric repeats were analyzed [39–41]. However, in this study, an opposite strategy is applied, the substitution of adenine for guanine (see Figure 1). The main aim is to achieve the total conversion of four human repeats to* Tetrahymena* repeats which retain the ability to form intramolecular G-quadruplex. Interestingly, G-rich repetitions containing imperfections were also found in the human and* Tetrahymena* genome; see Table 1 and Supporting Materials.

In this study, we examine the structures formed by the* Tetrahymena* telomeric sequence, dG_4_(T_2_G_4_)_3_, which differs from the human sequence by a single G-for-A replacement in each repeat [42]. Since Gs are essential for the formation of G-quadruplexes, we have systematically substituted each of the three adenines for guanines in the TTA loops of the G-quadruplex-forming sequence G_3_(T_2_AG_3_)_3_, thereby increasing the number of guanines by up to three guanines per oligonucleotide. Circular dichroism spectroscopy (CD) and polyacrylamide gel electrophoresis (PAGE) were used to observe the effect of base substitution (s) on the formation, thermal stability, and conformation of G-quadruplexes. The measurements were performed in the presence of both Na^+^ and K^+^ ions and with concentrations of either PEG-200 or acetonitrile at 0, 15, 30, and 50 wt% at different temperatures. In addition, the formation of G-quadruplex structures was verified and confirmed using Thiazole Orange (TO). TO is an excellent DNA fluorescent probe for DNA structural forms because of its high fluorescence quantum yield [43]. This ligand stabilizes the G-quadruplex structure and can also induce topological changes [44, 45]. The G-quadruplex-TO complex offers a characteristic profile of induced-circular dichroism spectrum in buffers containing sodium cations [44].

## 2. Materials and Methods

All experiments were carried out in a modified Britton-Robinson buffer (mRB), 25 mM phosphoric acid, 25 mM boric acid, 25 mM acetic acid, and supplemented by 50 mM of KCl or NaCl, PEG-200 (polyethylene glycol with an average molecular weight of 200) and acetonitrile (Fisher Slovakia); pH was adjusted by Tris to a final value of 7.0. Oligonucleotides with sequences shown in Table 1 were purchased from Metabion international AG. The lyophilized DNA samples were dissolved in double-distilled water prior to use to give 1 mM stock solutions. Single-strand DNA concentrations were determined by measuring the absorbance at 260 nm at high temperature (95°C).

### 2.1. CD Spectroscopy

CD and UV-vis spectra were measured using a Jasco model J-810 spectropolarimeter (Easton, MD, USA). The temperature of the cell holder was regulated by a PTC-423L temperature controller. Scans were performed over a range of 220–600 nm in a reaction volume of 300 *μ*l in a cuvette with a path length of 0.1 cm and an instrument scanning speed of 100 nm/min, 1 nm pitch, and 1 nm bandwidth, with a response time of 2 s. CD data represents three averaged scans taken at a temperature range of 0–100°C. All DNA samples were dissolved and diluted in suitable buffers containing appropriate concentrations of ions and dehydrating agent. The amount of DNA oligomers used in the experiments was kept close to 25 *μ*M of DNA strand concentration. The samples were heated at 95°C for 5 minutes then allowed to cool down to the initial temperature before each measurement. CD spectra are expressed as the difference in the molar absorption of the right-handed and left-handed circularly polarized light (Δ*ε*) in units of M^−1^·cm^−1^. The molarity was related to DNA oligomers. A buffer baseline spectrum was obtained using the same cuvette and subtracted from the sample spectra. The thermal stability of different quadruplexes was measured by recording the CD ellipticity at 295 and 265 nm as a function of temperature [14, 46]. The temperature ranged from 0 to 100°C, and the heating rate was 0.25°C/min. The melting temperature (*T*_*m*_) was defined as the temperature of the midtransition point. *T*_*m*_ was estimated from the peak value of the first derivative of the fitted curve. DNA titration was performed with increasing concentrations of TO. TO was solubilized in DMSO to reach a final concentration of stock solution of 10 mM. The concentration of DNA and TO in 1 mm quartz cell was 30 *μ*M and 0–200 *μ*M, respectively, and the increment of TO was ~67 *μ*M. Each sample was mixed vigorously for 3 min following the addition of TO; CD/UV spectra were measured immediately.

### 2.2. Electrophoresis

Samples consisting of 0.3 *μ*l of 1 mM stock solutions were separated using nondenaturing PAGE in a temperature-controlled electrophoretic apparatus (Z375039-1EA; Sigma-Aldrich, San Francisco, CA) on 15% acrylamide (19 : 1 acrylamide/bisacrylamide) gels. DNA was loaded onto 13 × 16 × 0.1 cm gels. Electrophoresis was run at 10°C for 4 hours at 125 V (~8 V·cm^−1^). Each gel was stained with StainsAll (Sigma-Aldrich). The gel was also stained using the silver staining procedure in order to improve the sensitivity of the DNA visualization [44].

### 2.3. Fluorescence Spectroscopy

The fluorescence spectra were acquired with a Varian Cary Eclipse Fluorescence Spectrophotometer at 22 ± 1°C which was equipped with a temperature-controlled circulator. A quartz cuvette with a 3 mm path length was used in all of the experiments. In the fluorescence measurements, the excitation and emission slits were 5 nm and the scan speed was 240 nm/min. 66 *μ*M of TO was titrated with DNA (3.3, 6.6, and 13.2 *μ*M) in a mRB buffer in both the presence and absence of monovalent metal cations. The molar ratios between DNA and ligand were 1 : 20, 1 : 10, and 1 : 5. The excitation wavelength was adjusted to 452 nm.

## 3. Results and Discussion

### 3.1. Sequence Design and CD Spectra

**Figure 2:**
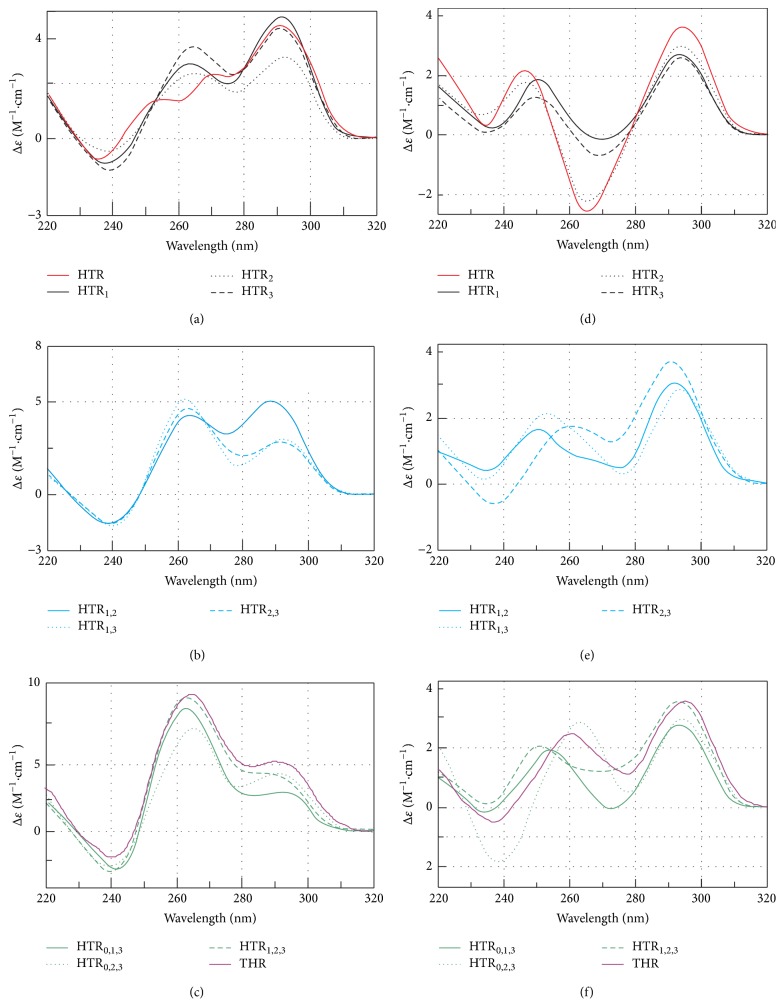
CD spectra of oligonucleotides used in this study in a 25 mM modified Britton-Robinson buffer (pH 7.0) in the presence of 50 mM KCl (a–c) and 50 mM NaCl (d–f). The HTR and THR spectra are shown in red and magenta, respectively. Each DNA sample was annealed at 95°C for 5 min and then allowed to cool for ~1 h to the initial temperature at which the sample was kept at the beginning of the measurement [14].

The sequence derived from human telomeric sequence d(G_3_(T_2_AG_3_)_3_) and substituted derivatives under different conditions are studied. The DNA sequences and the abbreviations used in this study are summarized in Table 1. Points 1, 2, and 3 indicate the positions of the base substitution in the first, second, and third loops of the HTR sequence, respectively. Point 0 indicates a flanking guanine at the 5′ end of the oligonucleotide, Figure 1. In the DNA oligonucleotides derived from HTR, the guanine (G)-for-adenine (A) in the TTA loop was substituted with the expectation that the modified sequences would retain the ability to form G-quadruplexes spontaneously, albeit with different topologies than those found in HTR sequences. The HTR derivatives were analyzed in the presence of both 50 mM NaCl and KCl, Figure 2. The first group represents oligonucleotides containing only single point mutations at different positions; HTR_1_, HTR_2_, and HTR_3_ (black lines in Figure 2). The second group represents oligonucleotides containing two point mutations (spectra indicated with blue in Figure 2). The first two loops were modified in HTR_1,2_, the first and last loops were changed in HTR_1,3_ and the second and third loops were modified in HTR_2,3_. Oligonucleotides HTR_0,1,3_, HTR_0,2,3_, and HTR_1,2,3_ contained three G-for-A substitutions (spectra in green). The spectrum and melting temperatures of the HTR_0,1,2_ sequence are very similar to those of the HTR_0,2,3_ sequence (not shown in this study), while the HTR_0,1,2,3_ sequence is equivalent to the THR sequence.

The substituted sequences were also compared with the unmodified HTR and THR sequences. In general terms, each of the guanine residues in any G-run could be involved in the formation of G-tetrads. In the case of the formation of three-layered G-tetrad quadruplexes, loop lengths were found to vary when the base substitution was introduced into the HTR sequence; loops could consist of three or four nucleotides depending on the location and number of substitutions. However, we cannot exclude the possibility of the formation of four-layered G-quadruplexes for sequences containing three substitutions, but it is important to note that such structures would have to consist of at least one heteronucleotide-tetrad in which adenine is also present. To date, the 3D structure of full-length THR sequences in presence of potassium has not been determined; the only facet of the structure which is known is the tetrameric G-quadruplex structure formed from four shorter sequences d(TTGGGGT) (PDB: 139D) [47]. This structure consists of four G-tetrads and cannot be stated as representing the real structure of a full-length oligonucleotide. Nevertheless, the 3D structure of THR has been ascertained only in the presence of sodium (PDB: 186D) [48]. This structure consists of three stacked G-tetrads, two edgewise loops, and one double-chain-reversal loop. Despite the fact that the sequences of THR and HTR differ at only one of the six nucleotides, their 3D topologies are quite different because HTR in sodium adopts a three-G-tetrad structure consisting of two edgewise loops and one central diagonal loop (PDB: 143D) [4]. However, the HTR sequence can also adopt a stable basket-type conformation in the presence of potassium consisting of only two G-tetrad layers (PDB: 2KF8) [31].

Several naturally occurring HTR sequences have been identified to date. Forms 1 (PDB: 2HY9) and 2 (2JPZ) consist of three G-tetrads, but the order of loops differs; HTR forms one double-chain-reversal and two edgewise loops in both forms [49, 50]. There is some similarity with THR G-quadruplexes which form in solution in the presence of sodium [48]. Form 3 is represented by a parallel G-quadruplex with three double-chain-reversal loops (PBD: 1KF1) [3]. Recently, Lim et al. have also confirmed the structure of a 27-nt HTR derivative in the presence of sodium which differs significantly from those mentioned above (PBD: 2MBJ) [1]. Although both known HTR structures solved in sodium possess the same relative strand orientations, they differ in the hydrogen-bond directionalities and in the loop arrangement. The 2MBJ structure again consists of two edgewise and one double-chain-reversal loops.

The sequence derived from the telomere of Oxytricha d[G_4_(T_4_G_4_)_3_] (PDB: 201D and 230D) adopts a structure with similar types of loops to those found in HTR in sodium; two edgewise and one central diagonal loops [4, 51, 52]. However, the Oxytricha sequence forms a four-layered G-tetrad quadruplex. At the time of writing, the solution structure of Oxytricha sequence d[G_4_(T_4_G_4_)_3_] in K^+^ containing solution had yet to be determined. The main reason for this could be the fact that this sequence and THR in the presence of potassium can adopt different topological forms which coexist in solution; additional bands are observed during electrophoretic separation [14]. Interestingly, the four-layered G-quadruplexes are very stable, exhibiting particularly high melting temperatures in the presence of potassium [14]. Recently, the structure of d(GGGGCC)_4_ in the presence of potassium has also been determined; the sequence contains cytosines instead of thymine residues and one 8-bromodeoxyguanosine (PDB: 2N2D) [53]. The G-quadruplex structure adopted by this sequence could be closely related to that of THR in potassium. This antiparallel structure is composed of four G-quartets which are connected by three edgewise C-C loops. CD spectra results show many signatures in common with the THR sequence. One of the cytosines in every loop is stacked upon the G-quartet; an arrangement which results is a very compact and stable structure. Similarly, the melting temperature of the structure is higher than 90°C.

It is generally accepted that CD spectroscopy is a very useful and cost-efficient method for offering a first glance at the architecture of folded G-quadruplexes. CD spectra of G-quadruplexes can be used to indicate whether the DNA has folded into a parallel or antiparallel conformation [36, 54].

Although there are up to 25 generic folding topologies of G-quadruplexes, it is possible to classify the structures into three groups based on the sequence of glycosidic bond angles adopted by guanosines of the G-quadruplex [55]. Group I consists of parallel G-quadruplexes with strands oriented in the same direction and with guanosines of the same glycosidic bond angles. Parallel G-quadruplexes (Group I) share the same characteristics irrespective of whether they contain three or four loops: an intense positive maximum at ~265 nm and minimum at ~240 nm. Groups II and III consist of antiparallel G-quadruplexes; Group II can be characterized by guanosines of glycosidic binding angles in orientations such as* anti-anti* and* syn-syn* and also* syn-anti* and* anti-syn*, while Group III consists of stacked guanosines of distinct glycosidic bonding angles. Antiparallel G-quadruplexes show a positive band at ~295 nm. Positive and negative CD signals at ~265 nm at ~240 nm, respectively, are characteristic for Group II, while Group III shows reverse peaks. In contrast, the CD spectra of high ordered G-quadruplex architecture of Group III forms exhibit negative and positive signals at 240 nm and ~265 nm, respectively [55].

CD profiles corresponding to distinct G-quadruplex conformations are determined empirically; therefore, the interpretation of CD spectra of unknown putative G-quadruplex sequences can be ambiguous. A number of other factors can also cause a degree of uncertainty over the evaluation of CD spectra, including, for example, the presence of mixed populations of various conformers and/or the presence of multimeric conformations in solution [9, 14, 44, 46].

CD measurements clearly show that the G-for-A substitutions had a considerable impact on the spectral profile of each sequence. The presence of the G-quadruplex scaffold formed from the unmodified HTR sequence is characterized by a positive peak at ~295 nm with two shoulders at around ~270 and ~250 nm in the presence of potassium (Figure 2(a), red line). According to CD spectra these signatures are characteristic for Group II antiparallel G-quadruplexes. This spectrum is indicative of the formation of a two-layered basket-type structure [31, 55]. The HTR sequence adopts a clear antiparallel G-quadruplex conformation of Group II type in the presence of sodium (Figure 2(d), red line). The structure is characterized by a large positive maximum at ~295 nm, a smaller one near ~245 nm, and a negative CD peak at ~265 nm. Previous studies have reported that these sequences form an intramolecular, basket-type antiparallel G-quadruplex [4]. Every sequence shows a clear peak at ~295 nm which is characteristic of an antiparallel G-quadruplex topology. The first set of oligomers with a single substitution per oligonucleotide in the presence of potassium shows two separated peaks at ~295 and ~265 nm; the signal is dominant at 295 nm (spectra shown in black). However, THR and HTR derivatives containing one or more G-for-A substitutions in the presence of potassium show an increase of the peak at ~265 nm (Figures 2(b) and 2(c)). This indicates the coexistence of more than one topological structure, that is, both parallel and antiparallel configurations; see also the electrophoretic results in Figure 7. The structural polymorphism was seen to increase with increasing numbers of Gs in the DNA sequence. The CD signal at ~265 nm (spectra shown in green) was predominant for oligonucleotides containing three substitutions (Figure 2(c)).

In the presence of sodium, only the HTR_2_ sequence with a substitution in the second loop exhibited a CD spectrum identical to that of HTR, although even this correspondence displayed lower amplitudes (spectra in dotted black in Figure 2(d)). HTR_1_ and HTR_3_ sequences with substitutions in the first and third loop, respectively, also displayed a positive maximum at 295 nm, but the negative peaks at 265 nm were shallower and slightly shifted towards longer wavelengths in comparison to the results of the unmodified sequence. Despite these differences, they are nonetheless likely to form G-quadruplexes of Group III. Only the CD spectra of the THR sequence shows signatures of Group II types.

Samples in the second group exhibited a positive maximum at ~295 nm with a slight shift to lower wavelengths in the case of HTR_1,2_ and HTR_1,3_ (Group III, spectra shown in blue in Figure 2(b)). These two sequences displayed a lack of a negative peak at 265 nm, and the smaller positive peak at around 245 nm was shifted slightly to longer wavelengths (Figure 2(e)). HTR_2,3_ shows a negative signal at ~245 nm and a positive signal at 265 and 295 nm, results which are indicative of the formation of Group II antiparallel G-quadruplexes.

The CD spectra of HTR_0,2,3_ are close to those of Group II G-quadruplexes while the CD of HTR_0,1,3_ and HTR_1,2,3_ resemble those of Group III G-quadruplexes. All samples with three mutations exhibited a positive maximum at ~295 nm . HTR_0,1,3_ exhibits negative signals at 235 and 275 nm in the presence of sodium (Figure 2(f)), while HTR_0,2,3_ shows positive signals at 265 and 295 nm.

In general, all the modified sequences in the presence of both Na^+^ and K^+^ were seen to differ to some degree from the HTR spectrum and were also found to differ from each other. The varying CD spectral profiles from sample to sample are a result of slight changes in G-quadruplex topology. However, it was not possible to determine either the group or structure of the G-quadruplexes with any degree of certainty on the basis of CD spectral profiles alone due to the coexistence of various topological forms, a finding which was confirmed by the electrophoretic results discussed in Section 3.5.

### 3.2. CD Spectra in the Presence of PEG-200 and Acetonitrile

**Figure 3:**
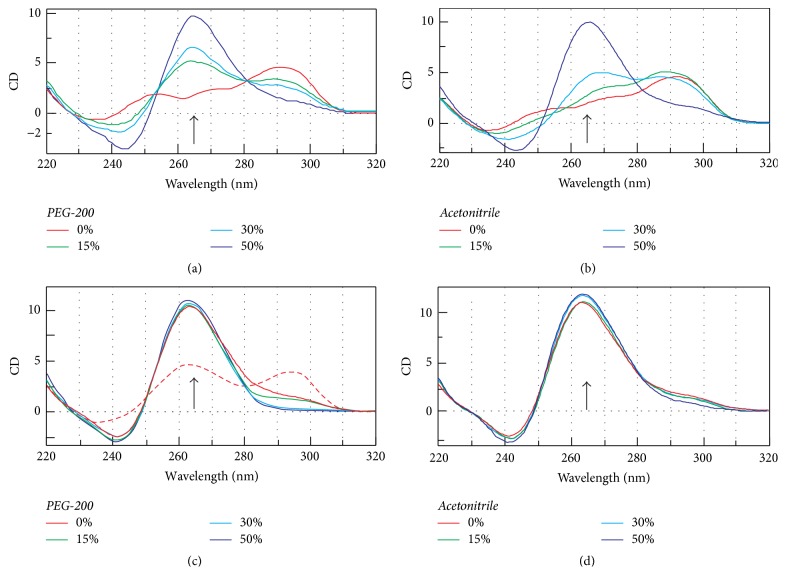
CD spectra of HTR (a and b) and THR (c and d) oligomers in a 25 mM mBR buffer (pH 7.0) in the presence of 50 mM KCl (red lines). The samples contain either PEG 200 or acetonitrile; 15% (v/w) (green lines), 30% (v/w) (light blue lines), and 50% (v/w) (dark blue lines). The increase in the magnitude of CD signals at 265 with increasing concentrations of dehydrating agent is marked by an arrow in both panels. Each DNA sample was annealed at 95°C for 5 min and then allowed to cool overnight at 4°C. The dashed red line represents the spectrum of THR without annealing.

In the presence of K^+^, the dehydrating agent PEG-200 is known to induce a conformational change of telomeric G-quadruplexes, primarily the transition from an antiparallel structure to a parallel arrangement [2, 9, 11, 13, 14, 56]. Therefore, the influence of PEG-200 and another dehydrating agent acetonitrile on CD spectral results and the stability of HTR derivatives were also investigated. The representative CD spectra of HTR and THR in the presence of different concentrations of both dehydrating agents (15, 30, and 50 wt%) and 50 mM KCl are shown in Figure 3. Both types of DNAs were found to form G-quadruplex structures with a propeller-like parallel arrangement in the presence of K^+^. However, when the sequences were studied in the presence of sodium with no potassium present, no structural conversions were observed; this finding remained constant for all of the studied HTR derivatives and THR. Interestingly, at a PEG-200 concentration of 50 wt% the positive peaks at 295 nm were found to disappear and a CD signal at 265 nm was recorded which was ~2-fold higher than without the presence of PEG-200. The same effect was observed for acetonitrile. This is an intrinsic property of any converted G-quadruplex molecule. In a recent study, our group presented a hypothesis which explains this fact; the CD signal depends on the number and orientation of stacked glycosyl bonds [9, 14, 57]. We have also previously shown that PEG-200 causes the dimerization of HTR [9]; therefore electrophoretic analysis of the sequences in the presence of PEG-200 was also performed, Figure 8.

The melting temperature of HTR and the vast majority of G-quadruplex structures are known to increase in the presence of PEG-200. In order to verify this fact, the melting temperatures were determined in the presence of PEG-200 on the basis of CD melting curves. The results are summarized in Table 2 and clearly confirm that PEG-200 increases the melting temperatures of HTR derivatives. In a methodology, which has been used in our previous studies, dual wavelength measurements were performed for cases in which the spectra displayed peaks at both 295 and 265 nm, respectively [9, 11]. A *T*_*m*_ of 63.2°C was obtained in a mRB buffer containing 50 mM KCl, as compared to a value of 50.4°C in a buffer with 50 mM NaCl for HTR at 295 nm. The overall picture which emerges from the thermodynamic data is that the stability of G-quadruplexes of HTR derivatives increases with increased numbers of G-for-A substitutions in both Na^+^ and K^+^ solutions. The lowest *T*_*m*_ value of HTR was recorded in both 50 mM KCl and 50 mM NaCl. The *T*_*m*_ of HTR derivatives was found to be higher in KCl than in NaCl. All of the studied sequences show a higher *T*_*m*_ value in the presence of both dehydrating agents. The results indicate that PEG-200 stabilizes G-quadruplexes with or without the A-for-G mutation. The proposed melting temperatures summarized in Table 2 clearly demonstrate that both the number of guanine residues in a G-tract and the nature of the stabilizing ion are important determining factors in the thermal stability of G-quadruplexes.

### 3.3. Titration Measurements

Our group has recently developed a new experimental methodology for the identification of G-quadruplex forming sequences using the cyanine dye Thiazole Orange (TO). TO is an excellent DNA fluorescent probe for various structural motifs due to its high fluorescence quantum yield [58]. This experimental technique can also be used to investigate the hypothesis that HTR derivatives adopt G-quadruplex conformations. TO interacts with various DNA secondary structures, but it has a stronger binding affinity to triplex and G-quadruplex structures than to other structural motifs [43, 45]. Although TO is optically inactive, TO-quadruplex complexes are chiral and display a unique profile of the induced CD (ICD) spectrum in the visible region [44]. Recently we have described the common ICD features shared by many different G-quadruplex structures. The results of TO-quadruplex interaction are the positive peaks at 265 and 295 nm (UV range), and the three peaks in the visible region at ~512, ~492, and ~473 nm in the solution either without the presence of metal cations or in presence of Na^+^ [44]. TO facilitates the formation of G-quadruplex structures even without the presence of other cations, but the adopted topology induced with TO can vary in comparison with the presence of sodium or potassium in solution; the CD profile in the UV region can be different. A completely different ICD profile of the TO-DNA complex was observed for sequences unable to adopt G-quadruplex structure [44]. However, other G-quadruplex ligands tested in our laboratory were not suitable for this purpose and provided ambiguous results; for example, Thioflavin T, porphyrin derivatives, Hoechst 33342, and Hoechst 33258. This methodology is intended to be used as a supplementary technique because it extends the possibilities of basic spectral methods in terms of distinguishing G-quadruplex structures without the use of more expensive and time-consuming methods. ICD monitoring can be applied in different conditions, but it is the most sensitive in solutions without the presence of metal cations; it can also be applied with slightly reduced sensitivity in solutions containing Na^+^ or low concentrations of K^+^ (<5 mM). It should be noted here that the interpretation of ICD profile at higher concentration of K^+^ is by no means unambiguous. Nevertheless, we also performed the titration experiments in the presence of 50 mM KCl because this condition is more biologically relevant.

**Figure 4:**
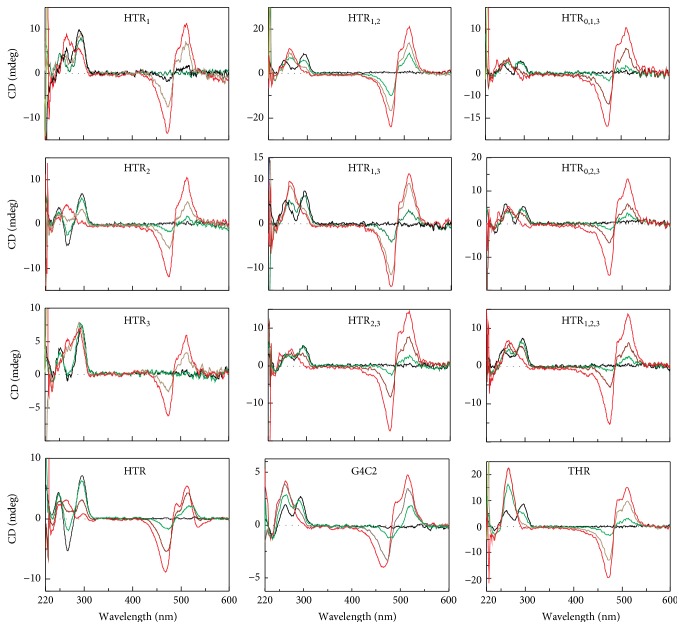
The CD titration spectra of 27 *μ*M DNA sample with TO; 0, 2.5, 5, and 7.5 molar equivalents of TO represent by black, green, brown, and red lines, respectively. Each sample was measured in a modified 25 mM mBR buffer containing 50 mM NaCl.

The results of titration analysis in the presence of 50 mM NaCl are shown in Figure 4. The ICD results display the expected positive signals at ~495 and ~510 nm and negative signals at ~475 nm, signatures which are characteristic for G-quadruplexes. In addition, the G4C2 sequence was analyzed because of its 3D structure in solutions containing potassium. As expected, this oligonucleotide was also found to form G-quadruplexes under the given conditions. Signals corresponding to those of antiparallel G-quadruplex structures were also clearly detected in the UV region. By increasing the concentration of TO, the signals at 295 and 265 nm were seen to decrease and increase, respectively, phenomena which are indicative of a conversion from antiparallel to parallel folding. This effect was also observed under the influence of PEG-200, Figure 3.

**Figure 5:**
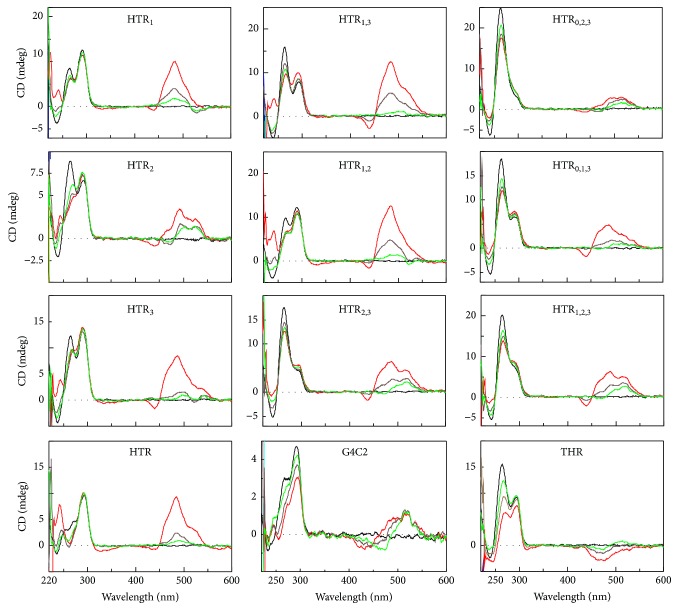
CD titration spectra of 27 *μ*M DNA sample with TO. 0, 2.5, 5, and 7.5 molar equivalents of TO represented by black, green, brown, and red lines, respectively. Each sample was measured in a modified 25 mM mBR buffer containing 50 mM KCl.

As was noted above, titration measurements in 50 mM KCl were also performed, Figure 5. The signals observed in the UV region clearly suggest that G-quadruplex structures were formed in the presence of potassium, but the effect of structural conversion was significantly suppressed. An intensive ICD signal with a maximum of around 500 nm is known to correspond to the formation of complexes between DNA and ligands. However, there was a distinct lack of any of the clear common features which are typically observed for profiles obtained in the presence of sodium. Interestingly, the ICD of the THR sequence was inverted, and therefore we suggest that the binding mode of TO with THR is different from that with HTR derivatives. Another explanation is that THR forms at least two distinct topological conformations in solution and that one of these forms can bind with TO more effectively. As was reported in our previous study, THR forms at least three different structures in the presence of potassium [14]; we therefore decided to verify this hypothesis using electrophoretic separation.

It is important to exclude the potential side effect of using DMSO during TO titration experiments. The stock solution of TO contains DMSO, a polar aprotic solvent which may produce an effect similar to that of PEG-200 [56]. The concentration of DMSO used in our experiments did not exceed 4.5 wt%. In order to eliminate the dehydrating effect DMSO may cause in TO titration experiments, titration analysis was also performed in the presence of DMSO alone. However, no significant effect was observed at concentrations lower than 5 wt% in the absence of Na^+^, K^+^, and ions. Nevertheless, the presence of DMSO in solution containing K^+^ could explain the slight differences in ICD profiles at concentrations of K^+^ greater than 5 mM.

### 3.4. Fluorescence Analysis

**Figure 6:**
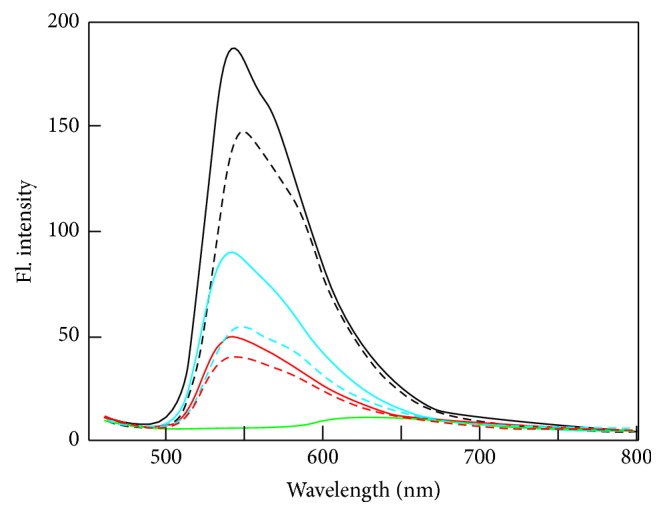
Fluorescence emission spectra (a.u.) of TO in the presence of HTR (solid lanes) and THR (dashed lines) in mRB buffer. The spectra without the presence of metal cations with 50 mM NaCl and 50 mM KCl are represented by black, blue, and red lines, respectively. The molar ratio of DNA : ligand is 1 : 5. Fluorescence emission of TO is shown in green.

DNA-TO complexes display a clear but wide absorption at around 500 nm. A single positive peak of TO was observed at 452 nm and this wavelength was used for the excitation of the DNA-TO complex. The fluorescence spectra of the HTR and THR sequences are shown in Figure 6. The measurements were performed in three different environments: (i) a mRB buffer without metal cations, (ii) a mRB buffer supplemented with 50 mM NaCl, and (iii) mRB supplemented with 50 mM KCl. For both oligomers, the fluorescence enhancement achieved the highest yield in the solution without metal cations. The fluorescence yield of HTR was greater than the yield of THR in all three of the tested conditions. The goal of this experiment was to demonstrate that the profile of fluorescence is not greatly dependent on the sequence of DNA oligonucleotide for this ligand. The fluorescence enhancement of TO can induce the formation of any type of G-quadruplex structure.

### 3.5. Electrophoresis in the Presence of Na^+^, K^+^, and TO

**Figure 7:**
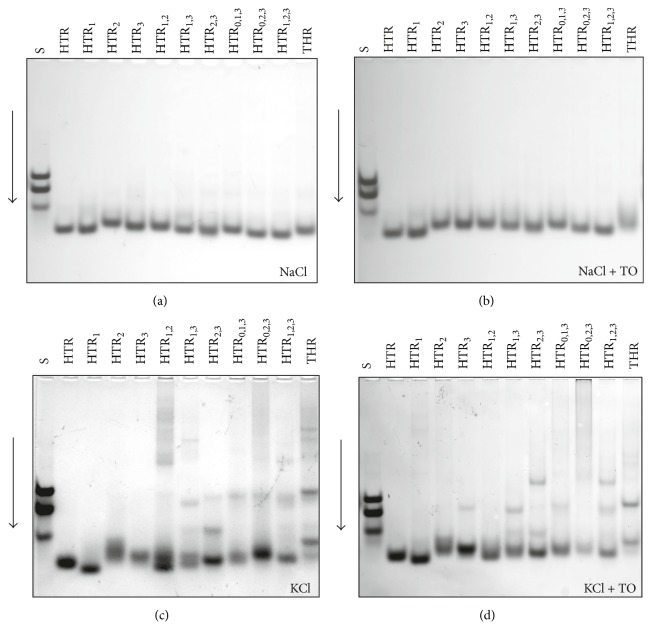
Electrophoretic records of studied DNA oligonucleotides. Electrophoretic gel and buffer contained 50 mM NaCl (a, b) and 50 mM KCl (b, d), gels in (b) and (d) also contained 30 *μ*M TO. 0.4 *μ*L of DNA from 1 mM stock solution was applied to each electrophoretic well (~3 *μ*M). The S-line represents the mobility of the mixture of oligonucleotides: d(AC)_9_, d(AC)_14_, and d(AC)_18_.

Nondenaturing polyacrylamide gel electrophoresis (PAGE) is an accessible technique which is used to supplement spectroscopic data when the presence of multiple species of G-quadruplexes cannot readily be identified based on CD spectra alone. The mobility of the DNA sample depends on many different factors, including conformation, charge, and molecular mass. Electrophoretic separation can provide valuable information about the molecularity of G-quadruplexes. Intramolecular G-quadruplexes have a compact structure and thus migrate faster through a cation-containing gel than their linear counterparts, while intermolecular G-quadruplexes migrate more slowly due to their higher molecular weight [9, 14, 44]. Oligomers d(AC)_9_, d(AC)_14_, and d(AC)_18_ were used as standards due to their lack of secondary structures. These standards served as benchmarks in comparing the mobility of different electrophoretic patterns. Since none of the sequences used were longer than 22 nt., the oligonucleotides which were observed to have migrated faster than d(AC)_9_ could be identified as having formed intramolecular G-quadruplexes. It is also reasonable to assume that oligonucleotides which moved more slowly or at a similar speed to d(AC)_18_ had adopted high-order G-quadruplex structures. Figure 7 shows the electrophoretic records of native 15% polyacrylamide gels illustrating the relative mobilities of the oligomers in the presence of 50 mM NaCl and KCl at 10°C (Figures 7(a) and 7(c)). In addition, the corresponding electrophoretic results, where the gels and loading buffers contain 2 molar equivalents of TO, are shown in Figures 7(b) and 7(d). In general, some clear trends emerge. Gel electrophoresis performed in the presence of sodium shows that all of the oligonucleotides had moved in one bulk, with single bands migrating faster than d(AC)_18_ in each column. This effect was also observed when TO was present in the gel. These results indicate that all DNA oligonucleotides form antiparallel intramolecular G-quadruplexes under these conditions. These results agree with the results obtained by CD spectroscopy. It is important to note that intramolecular structures had formed exclusively in the presence of sodium despite the introduction of mutations in HTR sequences increasing the possibility of the formation of different topologies of G-quadruplexes. The electrophoresis did not reveal any significant anomalous mobility of oligomers; sequences with the same length were found to move more or less equally.

In contrast to sodium, the presence of potassium led to the formation of both intra and intermolecular arrangements (Figures 7(b) and 7(d)). In the first group, the HTR_1_ quadruplex with one substitution in the first loop exhibited the fastest migrating band in comparison to that of HTR. A single smear band was also observed for the HTR_2_ sequence. Smears typically arise when two distinct conformers can be formed; a slow isomerization between the two conformers during the electrophoretic separation is the main source of band smearing. The mobility of the HTR and HTR_3_ sequences with substitutions in the third loop is similar. The oligonucleotides containing two substitutions per oligomer displayed high levels of polymorphism. These oligonucleotides form several coexisting conformers because each line contains several bands moving at different rates. Interestingly, the HTR_1,2_ and HTR_1,3_ sequences displayed two faster well-recognized bands, results which correspond to the formation of intramolecular conformers, and slower bands representing multimeric structures. The addition of TO also caused the fastest conformers to coalesce and the slowest structures to diminish. HTR_2,3_ produced a faster intra- and slower intermolecular species (dimer and tetramer). Surprisingly, the oligonucleotides with three substitutions per oligomer were found to be slightly less polymorphic in comparison with the sequences containing two substitutions, displaying only bands with lower magnitudes corresponding to the formation of multiple-molecular G-quadruplexes in the case of the HTR_0,1,3_ and HTR_1,2,3_ sequences. TO was found to exert only a limited effect on the multimeric forms of these oligonucleotides.

### 3.6. Electrophoresis in the Presence of PEG-200

**Figure 8:**
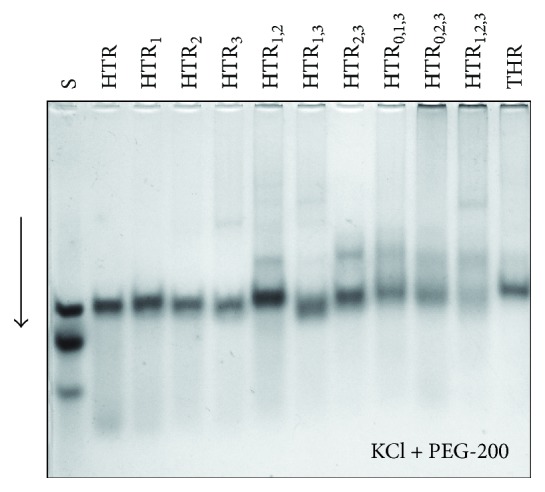
Electrophoretic record of DNA oligonucleotides in the presence of PEG-200. Electrophoretic gel and buffer contained 50 mM KCl. DNA samples were heated to 95°C for 5 min, slowly cooled, and then loaded into the electrophoretic wells. 0.4 *μ*L of DNA from 1 mM stock solution was applied to each electrophoretic well (~3 *μ*M). The loading buffer contained 50 wt% PEG-200.

The dependence of HTR dimerization on PEG 200 concentration has been analyzed in previous studies [9]. The formation of both intermolecular dimers and intramolecular monomers was observed in the buffer containing a PEG-200 concentration of 15% wt. The HTR derivatives containing 2 and 3 substitutions were seen to convert readily to slower migrating dimeric structures even at lower concentrations of PEG-200. At a PEG-200 concentration of 50 wt% and 50 mM KCl, the complete structural conversion to a parallel dimeric G-quadruplex was induced, Figures 3 and 8. This effect was not observed in buffers that did not contain potassium [9, 14]. CD measurements at 50 wt% PEG-200 showed no signal at ~295 nm. Based on our previous studies, intermolecular species which migrate more slowly are indicative of the formation of dimers [2, 9, 14]. The 3D structure of HTR containing a flanking sequence in an analogical condition has been determined using NMR [11]. The results show an intramolecular parallel G-quadruplex structure (PDB: 2LD8), but the overhanging nucleotides can cause a steric hindrance for the dimerization of this structure.

## 4. Conclusion

In this study, we clearly demonstrate that increasing the number of guanines in the loop regions of HTR sequences supports the formation of G-quadruplex structures. Any substitution of A-for-G increases the melting temperature, while the introduction of several substitutions was found to facilitate the coexistence of several conformers in the presence of potassium. The systematic introduction of these substitutions finally leads to the formation of sequences which occur in the* Tetrahymena* telomere. In addition, similar sequences were also found in the human genome. These findings raise an interesting point. Why does the* Tetrahymena* telomere require sequences which can adopt such highly stable G-quadruplex structures? In general, very stable G-quadruplexes are usually a source of problems in cells during the life cycle of an organism. The THR sequence is more polymorphic than HTR; it forms two different monomeric and one dimeric conformers as has been shown here and in our previous studies [14]. Our analysis focused on sequences consisting of four G-runs without any overhanging nucleotides at both termini; this type of arrangement is not an ideal model for extrapolation to natural telomeric repeats which typically consist of tens to thousands repeats.

Our results demonstrate that all HTR derivatives including THR can be converted from antiparallel to parallel folds in the presence of potassium and PEG-200. ICD spectra indicate that the binding mode of TO with THR in the presence of KCl might be different from those observed for HTR derivatives, and this is a finding which could also be important for other molecules recognizing the THR structure in nature. It suggests that the structure of THR shows some structural features which are different from those of HTR and HTR derivatives in the presence of potassium. Confirmation of the biological significance of this fact remains an open topic.

## Figures and Tables

**Table 1:** DNA oligodeoxynucleotides used in this study and their occurrence in human and *Tetrahymena* genomes.

Name	*ε* ^a^ (mM^−1^ cm^−1^)	Sequence^b^ (5′ → 3′)	Occurrence in genome (ID)	
			Human	Tetrahymena
HTR	225.4	GGGTTAGGGTTAGGGTTAGGG	Very high	-
HTR_1_	222.1	GGGTT**G**GGGTTAGGGTTAGGG	NG 054915.1	M11627.1.1
HTR_2_	222.1	GGGTTAGGGTT**G**GGGTTAGGG	NG 029533.1	-
HTR_3_	222.1	GGGTTAGGGTTAGGGTT**G**GGG	NT 187653.1	-
HTR_1,2_	218.8	GGGTT**G**GGGTT**G**GGGTTAGGG	NW 003571049.1	M11627.1
HTR_1,3_	218.8	GGGTT**G**GGGTTAGGGTT**G**GGG	NC 018926.2	M11627.1
HTR_2,3_	218.8	GGGTTAGGGTT**G**GGGTT**G**GGG	NC 018914.2	M11627.1
HTR_0,1,3_	230.0	**G**GGGTT**G**GGGTTAGGGTT**G**GGG	-	M11627.1
HTR_0,2,3_	230.0	**G**GGGTTAGGGTT**G**GGGTT**G**GGG	NC 018930.2	M11627.1
HTR_1,2,3_	215.4	GGGTT**G**GGGTT**G**GGGTT**G**GGG	NC 018912.2	AH001112.2
THR	213,4	GGGGTTGGGGTTGGGGTTGGGG	NG 034020.1	Very high
G4C2	204,4	GGGGCCGGGGCCGGGGCCGGGG	NG 052810.1	-
(AC)_9_	171.9	(AC)_9_	ND	ND
(AC)_18_	342,9	(AC)_18_	ND	ND

^a^Milimolar extinction coefficient at 257 nm. ^b^Base modifications are underlined. ND: not determined.

**Table 2:** Influence of PEG-200 on the melting temperatures of DNA oligomers in the presence of potassium and sodium ions.

Oligomer	*T*_*m*_ (°C) in 50 mM KCl + PEG-200				*T*_*m*_ (°C) in 50 mM NaCl + PEG-200			
	0%	15%	30%	50%	0%	15%	30%	50%
	295/265	295/265	295/265	295/265	295/265	295/265	295/265	295/265
HTR	63.2/62.9	63.2/ND	78.8/ND	ND/89.8	50.4/ND	51.2/ND	52.8/ND	64.0/ND
HTR_1_	71.8/73.1	71.4/76.3	64.3/83.1	ND/>95	55.7/ND	58.9/ND	61.5/ND	64.4/ND
HTR_2_	65.2/65.9	64.2/72.1	ND/79.6	ND/>95	51.2/ND	54.3/ND	58.1/ND	62.7/ND
HTR_3_	67.8/68.6	69.3/75.3	ND/81.7	ND/>95	50.6/ND	54.3/ND	58.7/ND	64.8/71.6
HTR_1,2_	69.5/70.5	ND/76.9	ND/83.2	ND/>95	55.9/ND	59.1/ND	62.8/ND	65.3/72.1
HTR_1,3_	72.2/71.5	70.5/79.1	ND/86.4	ND/>95	53.7/ND	56.2/59.8	60.1/63.2	ND/73.5
HTR_2,3_	81.3/76.9	ND/79.7	ND/85.2	ND/>95	53.3/ND	57.3/ND	59.9/63.0	64.4/71.0
HTR_0,1,3_	73.0/80.0	ND/79.0	ND/87.9	ND/>95	52.5/ND	56.3/60.7	60.1/63.3	ND/72.7
HTR_0,2,3_	70.7/72.7	ND/77.6	ND/>95	ND/>95	51.8/54.5	56.0/51.1	59.4/60.2	ND/73.8
HTR_1,2,3_	73.9/77.8	ND/83.9	ND/88.6	ND/>100	56.4/ND	59.0/ND	61.2/64.0	ND/75.7
THR	83.1/ND	87.7/ND	>95	ND	57.4/ND	59.7/ND	ND/66.8	ND
